# A histone H3K9 methyltransferase Dim5 mediates repression of sorbicillinoid biosynthesis in *Trichoderma reesei*

**DOI:** 10.1111/1751-7915.14103

**Published:** 2022-08-03

**Authors:** Lei Wang, Jialong Liu, Xiaotong Li, Xinxing Lyu, Zhizhen Liu, Hong Zhao, Xiangying Jiao, Weixin Zhang, Jun Xie, Weifeng Liu

**Affiliations:** ^1^ Collaborative Innovation Center of Reverse Microbial Etiology Department of Biochemistry and Molecular Biology, College of Basic Medical Sciences Shanxi Medical University Taiyuan China; ^2^ Institute of Basic Medicine Shandong First Medical University&Shandong Academy of Medical Sciences Jinan China; ^3^ State Key Laboratory of Microbial Technology Institute of Microbial Technology, Shandong University Qingdao China

## Abstract

Sorbicillinoids (also termed yellow pigment) are derived from either marine or terrestrial fungi, exhibit various biological activities and therefore show potential as commercial products for human or animal health. The cellulolytic filamentous fungus *Trichoderma reesei* is capable to biosynthesize sorbicillinoids, but the underlying regulatory mechanism is not yet completely clear. Herein, we identified a histone H3 lysine 9 (H3K9) methyltransferase, Dim5, in *T. reesei*. *TrDIM5* deletion caused an impaired vegetative growth as well as conidiation, whereas the ∆*Trdim5* strain displayed a remarkable increase in sorbicillinoid production. Post *TrDIM5* deletion, the transcription of sorbicillinoid biosynthesis‐related (*SOR*) genes was significantly upregulated with a more open chromatin structure. Intriguingly, hardly any expression changes occurred amongst those genes located on both flanks of the *SOR* gene cluster. In addition, the assays provided evidence that H3K9 triple methylation (H3K9me3) modification acted as a repressive marker at the *SOR* gene cluster and thus directly mediated the repression of sorbicillinoid biosynthesis. Transcription factor Ypr1 activated the *SOR* gene cluster by antagonizing *Tr*Dim5‐mediated repression and therefore contributed to forming a relatively more open local chromatin environment, which further facilitated its binding and *SOR* gene expression. The results of this study will contribute to understanding the intricate regulatory network in sorbicillinoid biosynthesis and facilitate the endowment of *T. reesei* with preferred features for sorbicillinoid production by genetic engineering.

## INTRODUCTION

Fungi are potent producers of secondary metabolites (SMs) which are mainly divided into four groups including polyketides, nonribosomal peptides, alkaloids and terpenes (Katz & Baltz, 2016; Schmidt‐Dannert, 2015; Zhang et al., 2018b). In general, SMs are not directly essential for the basic growth or survival but have critical roles in some biological processes, such as signalling, development or cross‐talk with other organisms. In the post‐genomic era, an increasing number of genes for SM biosynthesis have been identified, and, intriguingly, they are frequently located in clusters in the fungal genomes (Khaldi et al., 2010). However, the majority of putative products remain unknown. Additionally, many gene clusters responsible for SM synthesis are silent or weakly expressed under laboratory culture conditions, which may not appropriately recapitulate the microbes' natural habitat (Hertweck, 2009), thereby restricting the large‐scale production and application of these products. Given this, various genetic manipulations to activate or enhance the expression of secondary metabolic gene clusters at the transcriptional level have been proven to be an effective strategy for obtaining novel natural products or achieving low‐cost synthesis and a large supply (Brakhage & Schroeckh, 2011).

Genome sequencing showed that gene clusters involved in secondary metabolism in fungi are usually adjacent to the telomere or heterochromatin region (Nierman et al., 2005). The epigenetic modifications (e.g. DNA methylation and histone methylation) influence the expression of these clusters (Hertweck, 2009; Reyes‐Dominguez et al., 2010; Shwab et al., 2007). Consequently, silencing of secondary metabolic gene clusters could be reversed by relieving these repressive factors including the compact local chromatin conformation within which SM gene clusters reside. The absence of histone deacetylase (HDAC) HdaA in *Aspergillus nidulans* caused an upregulation in the expression level of secondary metabolic genes located in subtelomeric regions which resulted in increased production of their encoded SMs, such as sterigmatocystin (ST) and penicillin (PN) (Shwab et al., 2007). *Schizosaccharomyces pombe* Clr4 is a histone methyltransferase responsible for the mono‐, di‐ and tri‐methylation of H3K9 (Kusevic et al., 2017). Previous studies have shown that H3K9 methylation is closely linked with heterochromatin establishment which represents a repressive state of gene transcription (Audergon et al., 2015). Inactivation of ClrD, which is a homologue of *Sp*Clr4, led to increased ST production in *A. nidulans* (Reyes‐Dominguez et al., 2010). In *Saccharomyces cerevisiae*, the mono‐, di‐ and tri‐methylation of histone H3 lysine 4 (H3K4) are catalysed by the complex associated with Set1 (COMPASS). These three distinct modes of modification on H3K4 elicit different effects on gene transcription (Deshpande et al., 2022). The absence of *A. nidulans* CclA, which is a component of COMPASS, activated a cryptic secondary metabolic gene cluster (Bok et al., 2009). Targeting HDAC or DNA methyltransferase (DNMT) activity with specific chemical agents has been proven to be effective for provoking changes in the profile of fungal secondary metabolism. For example, the treatment of *Trichoderma atroviride* with trichostatin A (TSA), an inhibitor of HDAC, altered the expression of the secondary metabolic gene and SM synthesis (Gómez‐Rodríguez et al., 2018). Similar results have also been reported in *Penicillium expansum*, *Aspergillus niger*, *Alternaria alternate*, *Cladosporium cladosporioides* and other fungi (Fisch et al., 2009; Henrikson et al., 2009; Liu et al., 2014; Williams et al., 2008). Moreover, the putative methyltransferase LaeA (a part of the velvet complex) is widely linked with changes at the chromatin level and shows a positive role in regulating the secondary metabolism of filamentous fungi including *Trichoderma* genus, although its precise substrate remains unknown (Bayram et al., 2019; Bok & Keller, 2004; Ding et al., 2020; Karimi‐Aghcheh et al., 2013a, 2013b). The *laeA* knockout resulted in noticeable ST loss and increased H3K9 methylation in the ST cluster (Bok et al., 2006). Further deletion of *hdaA* or *clrD* in the ∆*laeA* background partially restored ST production (Reyes‐Dominguez et al., 2010; Shwab et al., 2007). The above discussion, thus, indicated that secondary metabolism is regulated at the chromatin level by a complicated molecular mechanism in fungi.

Sorbicillinoids belong to the hexaketide metabolites and more than 90 types of sorbicillinoids have been identified from either terrestrial or marine fungi since their first discovery in 1948 (Meng et al., 2016). Sorbicillinoids show various inherent biological activities. For example, some compounds isolated from a sorbicillinoid mixture displayed more robust inhibitory activity against α‐glycosidase than acarbose, suggesting their potential role to treat diabetes (Pang et al., 2021). Sorbicillinoids also displayed highly effective photoinactivation activity for gram‐positive bacteria as a photosensitizer under irradiation with a nontoxic dose of UV light (Yang et al., 2020). *T. reesei* is an industry‐scale producer of cellulases and hemicellulases (Bischof et al., 2016; Zhang et al., 2018a). The fermentation processes of *T. reesei* are accompanied by sorbicillinoid secretion, which complicates the downstream process and increases the costs (Derntl et al., 2016; 2017a). Thus, eliminating sorbicillinoid secretion during cellulase production or developing a high sorbicillinoid production strain based on its biosynthesis mechanism will endow *T. reesei* with preferred features for industrial applications.

The *T. reesei SOR* gene cluster, located approximately on 30 kb chromatin region, contains a reducing polyketide synthase (PKS)‐encoding gene, *SOR1*; a nonreducing PKS‐encoding gene, *SOR2*; a flavin adenine dinucleotide (FAD)‐dependent monooxygenase‐encoding gene, *SOR3*; a FAD/flavin mononucleotide (FMN)‐containing dehydrogenase‐encoding gene, *SOR4*; a short‐chain dehydrogenase/reductase‐encoding gene, *SOR5*; a major facilitator superfamily (MFS) transporter‐encoding gene, *SOR6*; and two transcription‐factor‐encoding genes, *YPR1* and *YPR2* (Derntl et al., 2016; 2017a; Li et al., 2018). The absence of Ypr1, which is a potent activator of the *SOR* gene cluster, abolished sorbicillinoid synthesis. Ypr2 showed an inhibitory effect on *YPR1* expression, which may provide a negative feedback mechanism to ensure an appropriate amount of sorbicillinoids exists (Derntl et al., 2016). Previous studies have shown that *T. reesei* orthologues of velvet protein complex modulated sorbicillinoid synthesis (Aghcheh et al., 2014; Karimi‐Aghcheh et al., 2013a; Seiboth et al., 2012). Recently, our studies suggested that chromatin remodelling factor Isw1 participated in the regulation of sorbicillinoid synthesis in *T. reesei* (Cao et al., 2022). Otherwise, little evidence has been found for *T. reesei SOR* gene cluster regulation, especially at the chromatin level, to guide sorbicillinoid biosynthesis.

In this study, we identified *T. reesei* H3K9 methyltransferase Dim5, the orthologues of which are associated with heterochromatin formation in eukaryotic cells (Akoury et al., 2019). *DIM5*‐null *T. reesei* exhibited impaired vegetative growth and conidiation. However, a significant increase was found in sorbicillinoid biosynthesis after *TrDIM5* deletion. Compared with the control strain, the expression of *SOR* genes was upregulated in the ∆*Trdim5* strain, and this upregulation depended on Ypr1. ChIP assays showed that *Tr*Dim5 specifically introduced the repressive H3K9me3 modification into the *SOR* gene promoters, and this modification was antagonized by Ypr1. Further analyses of the chromatin status at *SOR* promoter regions revealed that a relatively more open chromatin occurred in a Ypr1‐dependent manner in the absence of *Tr*Dim5. These findings will contribute to engineering the *T. reesei* strain to obtain low‐cost raw material for developing sorbicillinoid‐based products.

## EXPERIMENTAL PROCEDURES

### Strains and culture conditions

*Escherichia coli* DH5α cells were cultured in LB medium at 37°C and were used for plasmid construction. Throughout the study, QM9414 (ATCC_26921) and its derivative QM9414‐Δ*pyr4* (Wang et al., 2018) were used as control and parental strains, respectively. All *T. reesei* strains were maintained on malt extract agar for conidia production. For sorbicillinoids production, *T. reesei* strains were pre‐grown in Mandels–Andreotti (MA) medium with 1% (v/v) glycerol as a carbon source on a rotary shaker (200 rpm) at 30°C for 48 h, as previously described (Wang et al., 2019a; Zhou et al., 2012). Mycelia were harvested by filtration and washed twice with MA medium without carbon source. An equal amount of mycelia was then transferred to a fresh MA medium containing 1% (w/v) Avicel or glucose, and incubation was continued for the indicated time periods. Uridine at a final concentration of 10 mM was added for *T. reesei* cultivation when necessary.

### Plasmid and recombinant *T. reesei* strain construction

Genomic DNA was extracted from *T. reesei* cells using the Fungal DNA Kit (Omega). To delete *TrDIM5* (*Trire2_111216*), two DNA fragments corresponding to approximately 2.1 kb of *TrDIM5* up‐ and downstream non‐coding regions were amplified from genomic DNA and ligated into pDONOR*pyr4* (Zhang et al., 2013) via BP‐cloning to yield the vector pDONOR*pyr4‐*∆*Trdim5*, which was then used to transform *T. reesei* QM9414‐Δ*pyr4* after linearization with I‐*Sce*I to result in the ∆*Trdim5* strain. Linearized pDONOR*pyr4‐*∆*Trdim5* was also transformed to Δ*Trypr1* (Zhang et al., 2021) to obtain Δ*Trypr1* & *Trdim5* strain. All the fungal transformations were performed as described by (Penttilä et al., 1987). The number of copies of the knockout cassette in mutant strains was confirmed by genomic qPCR (Figure S2). All the primers used for plasmid construction and genomic qPCR are listed in Table S3.

### Vegetative growth, conidiation and sorbicillinoid production analyses

To analyse *T. reesei* vegetative growth in a solid medium, equal amounts of mycelia were inoculated on minimal media agar plates containing 1% (w/v) concentration of different carbon sources (glucose, cellobiose or lactose) and incubated at 30°C for 3 days. To determine the growth in liquid culture, an equal amount of mycelia was transferred to fresh MA with 1% (w/v) glucose or 1% (w/v) Avicel as the sole carbon source. At the indicated time points, mycelia cultured on glucose were filtrated on filter paper, dried at 70°C for 48 h and then weighed. For Avicel cultures, the DNA content was measured to determine the growth due to the interference from insoluble Avicel (Figure S3).

For conidiation analysis, mycelia were inoculated on malt extract agar plates and incubated at 30°C for 5 days. The number of conidia was counted with a haemocytometer on an inverted optical microscope. To analyse sorbicillinoid production, 200 μl of culture supernatants of each strain grown on glucose or Avicel‐containing medium at different incubation time was collected and determined by measuring the absorbance at 370 nm using a microplate reader as previously described (Derntl et al., 2016; 2017b).

### Quantitative RT‐PCR (RT‐qPCR)

Total RNA was extracted using TRIzol reagent (Invitrogen) and cDNA synthesis was carried out using the PrimeScript RT reagent Kit (Takara). Quantitative PCR was performed on Applied Biosystems QuantStudio 3 (Thermo). Amplification reactions were performed using the SYBR Green Realtime PCR Master Mix (Toyobo) according to the manufacturer's instructions. Data analysis was performed using the comparative CT method and was normalized to an endogenous control (*ACTIN*) (Schmittgen & Livak, 2008). All primers used for amplification in qRT‐PCR assays are listed in Table S3.

### Chromatin accessibility real‐time PCR (CHART‐PCR)

CHART‐PCR assays were carried out as described by Mello‐de‐Sousa et al. with minor modifications (Mello‐de‐Sousa et al., 2014). Mycelia were harvested by filtration and ground to powder in liquid nitrogen. Portions (100 mg) of the powder were suspended in 1 ml of pre‐prepared nuclease digestion buffer (250‐mM sucrose, 60‐mM KCl, 15‐mM NaCl, 0.05‐mM CaCl_2_, 3‐mM MgCl_2_, 0.5‐mM DTT and 15‐mM Tris–HCl at pH 7.5), and 100 μl of the sample was incubated with 10 U of RNase‐free DNase I for 5 min at 37°C. The 100 μl of stop solution (40‐mM EDTA and 2% SDS) was added to the mixture followed by one round of phenol‐chloroform‐isopentanol (25:24:1) extraction and one round of chloroform‐isopentanol (24:1) extraction. Samples were then treated with 2 μl of 1 mg/ml RNase A for 15 min at 37°C. DNA was precipitated with 0.3‐M NaAc and two volumes of ethanol at −20°C for 2 h and resuspended in 50 μl of double‐distilled water. The relative abundance of the target genomic region in DNase I‐treated samples was determined by qPCR analysis. The amount of intact DNA of each sample was calculated by comparing it with a standard curve generated for each primer set using serial dilutions of undigested genomic DNA. The chromatin accessibility index (CAI) was defined as CAI = 1/Ds/Dc, where Ds is the amount of intact DNA detected for each target region and Dc is the amount of intact DNA detected for the promoter region of the housekeeping gene *ACTIN*. CHART‐PCR of R1‐R6 regions in the *SOR* gene cluster was performed in this study. R1 region was selected between the gene body of *SOR1* and *SOR2*; R3 region was selected between the gene body of *SOR6* and *YPR2*; R2, R4, R5 and R6 regions were selected from the promoters of *SOR3*, *SOR4*, *YPR1* and *SOR5*, respectively. Primers used for amplification in CHART‐PCR assays are listed in Table S3.

### Chromatin immunoprecipitation (ChIP)

ChIP assays were performed as previously described (Wang et al., 2019b; 2021). Briefly, the mycelia were incubated in MA medium containing 1% formaldehyde at 30°C for 10 min with shaking before the cross‐linking was quenched via adding 25 ml of 1.25‐M glycine. The mycelia were then collected, suspended in 50‐mM HEPES lysis buffer at pH 7.5 plus 150‐mM NaCl, 1‐mM EDTA, 0.5% Triton X‐100, 0.1% sodium deoxycholate, 0.1% SDS, 1‐mM PMSF, 1 μg/ml leupeptin and 1‐μg/ml pepstatin, and broken with glass beads (0.45 mm). Chromatin DNA was further sonicated to obtain sheared DNA fragments with an average size of approximately 500 bp. Immunoprecipitation with the antibody against H3 and H3K9me3 (Abcam) was performed (Cao et al., 2019). Quantitative PCR was performed on the precipitated chromatin DNAs. Relative enrichment of the DNAs was calculated as a percentage of the input DNA. All primers used for amplification in ChIP assays are listed in Table S3.

### Transcriptome sequencing and analysis

Total RNA was extracted using a Trizol reagent kit (Invitrogen) and its quality was assessed on an Agilent 2100 Bioanalyzer (Agilent Technologies) and checked using RNase free agarose gel electrophoresis. The mRNA was enriched by Oligo(dT) beads, fragmented into short fragments using fragmentation buffer and reverse transcripted into cDNA with random primers. Second‐strand cDNA was synthesized by DNA polymerase. Then, the cDNA fragments were purified with QiaQuick PCR extraction kit (Qiagen), end repaired, poly(A) added and ligated to Illumina sequencing adapters. The ligation products were size (~200 bp) selected by agarose gel electrophoresis, PCR amplified and sequenced using Illumina Novaseq6000 (Gene Denovo). The raw RNA‐seq data are available in Sequence Read Archive (SRA) database with accession number PRJNA822668. The expression analysis of all genes and DEGs with the threshold of |log_2_FC| > 1 and FDR <0.05 were presented in Data S1 and S2, respectively. Bioinformatics analysis was performed using Omicsmart, a dynamic and interactive online platform for data analysis (https://www.omicsmart.com).

### Protein sequence analysis

Amino acid sequences from *T. reesei* and other relevant species were obtained from the UniProt (https://www.uniprot.org/) or JGI (https://genome.jgi.doe.gov/) database. The phylogenetic analysis of *Tr*Dim5 was generated using the neighbour‐joining method with protein sequences aligned by ClustalW based on the JTT with MEGAX (Kumar et al., 2018). Statistical confidence of the inferred phylogenetic relationships was assessed by performing 1000 bootstrap replicates. The protein domain was predicted via InterPro (http://www.ebi.ac.uk/interpro/) database.

## RESULTS

### Identification of the *Sp* Clr4 orthologue in *T. reesei*

The posttranslational H3K9me3 modification is thought to be a heterochromatin marker which causes the transcriptional repression of local genes in *S. pombe* (Akoury et al., 2019; Farooq et al., 2019), but the physiological roles of H3K9me3 in *T. reesei* remain elusive. To identify the *T. reesei* H3K9 methyltransferase and investigate its biological functions, a homologous gene, *Trire2_111216* (hereafter named *TrDIM5)*, was found by searching the *T. reesei* genome (https://mycocosm.jgi.doe.gov/Trire2/Trire2.home.html) using *Sp*Clr4, (GenBank: CAA22283.1) as a query. Western blot analysis provided an evidence that *Tr*Dim5 is a crucial H3K9 methyltransferase in *T. reesei* (Figure S1).

**FIGURE 1:**
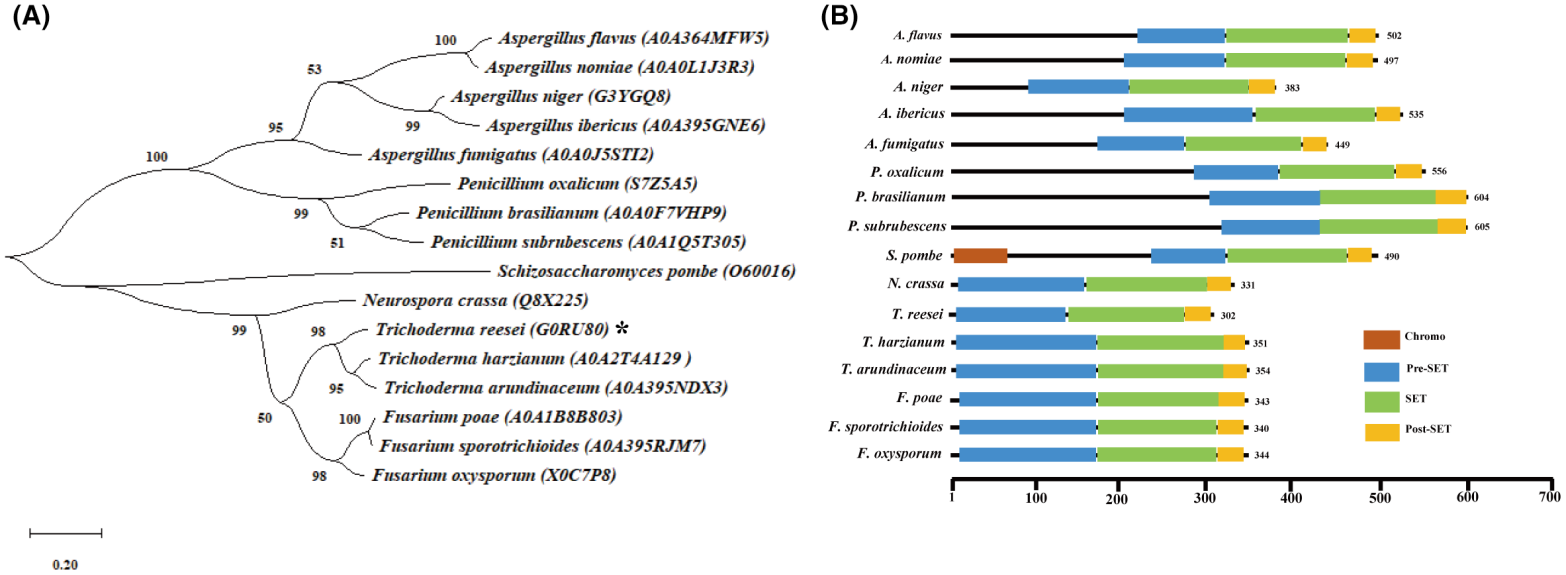
Phylogenetic analysis and domain prediction for *Tr*Dim5 and its orthologues. (A) The phylogenetic analysis of *Tr*Dim5 was generated using the neighbour‐joining method with protein sequences aligned by ClustalW based on the JTT with MEGAX. Statistical confidence of the inferred phylogenetic relationships was assessed by performing 1000 bootstrap replicates. *Tr*Dim5 is indicated with an asterisk. The entry numbers of these sequences in the UniProt database are included in brackets. (B) The conserved protein domains of *Tr*Dim5 and its orthologues were predicted via InterPro.

*TrDIM5* consists of four exons spaced by three introns encoding a protein with 302 amino acids (Schmoll et al., 2016). Protein BLAST analyses showed that Dim5 orthologues are widely distributed amongst filamentous *Trichoderma*, *Penicillium*, *Fusarium*, *Neurospora* and *Aspergillus* species, although they exhibit different amino acid lengths (Figure 1A, B). To analyse the phylogenetic relationship between Dim5 homologues from filamentous fungi and *Sp*Clr4, we built a phylogenetic tree using Dim5 orthologues from the above species as well as *Sp*Clr4. The results revealed that filamentous fungi Dim5 orthologues formed two distinct clusters from *Sp*Clr4. Amongst them, *Trichoderma* Dim5 showed a close evolutionary relationship with the orthologues from *Fusarium* and *Neurospora* species (Figure 1A).

Furthermore, domain analysis showed that all the Dim5 orthologues comprise three conserved domains, Pre‐SET, SET and Post‐SET, which may be responsible for the histone modification activity of these orthologues. Surprisingly, the N‐termini of Dim5 orthologues from *Aspergillus* and *Penicillium* species contain different lengths of amino acids without any known structural features that do not exist in the Dim5 orthologues from *Trichoderma*, *Fusarium* and *Neurospora* species. Moreover, unlike its counterparts in filamentous fungi, *Sp*Clr4 contains an N‐terminal chromodomain which is considered to be involved in the binding of methylated histones. Collectively, these analyses implied that some divergence in the function of Clr4 orthologues might occur during evolution not only between *S. pombe* and filamentous fungi but also amongst the filamentous fungal phylogenies (Figure 1B).

### *Tr* Dim5 facilitates vegetative growth and conidiation of *T. reesei*

**FIGURE 2:**
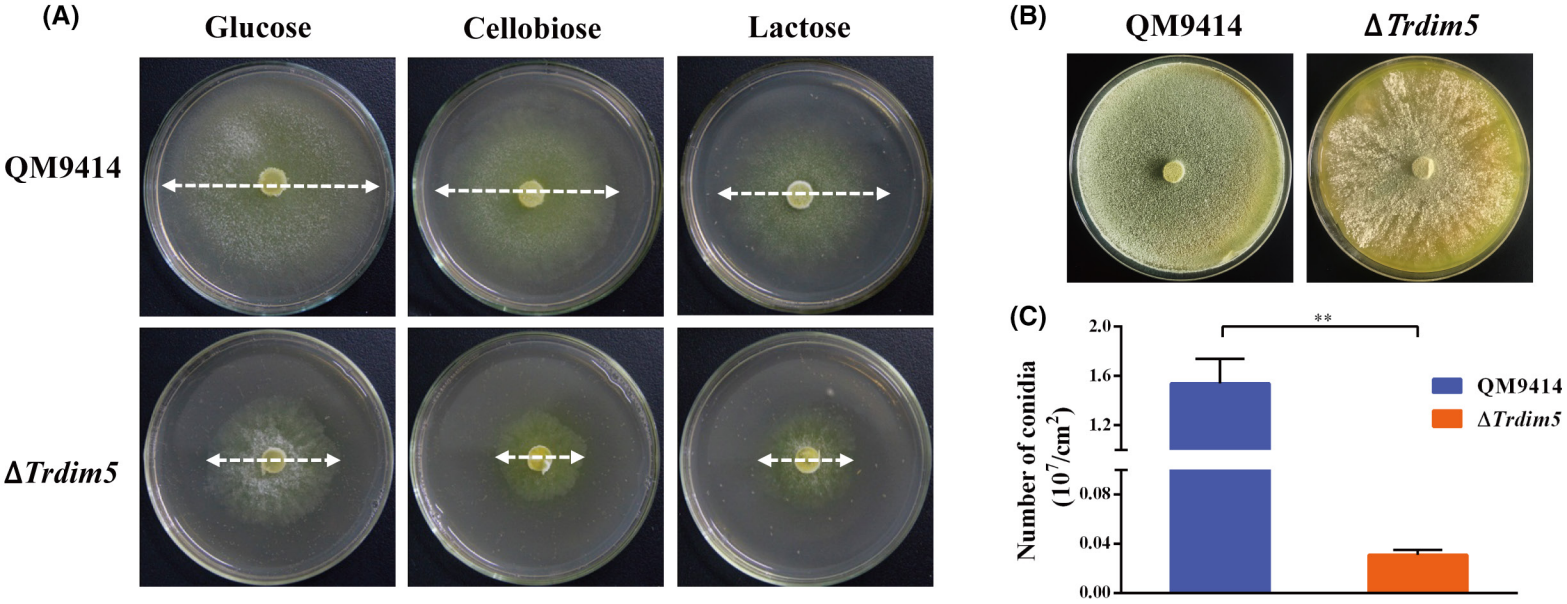
Disruption of *TrDIM5* compromised *T. reesei* vegetative growth and conidiation. (A) Growth of the QM9414 and ∆*Trdim5* strains on agar plates with various carbon sources at a final concentration of 1% (w/v) at 30°C for 3 days. (B) Conidiation analyses of QM9414 and ∆*Trdim5* strains on malt extract agar plates at 30°C for 5 days. (C) Quantification of conidia production by QM9414 and ∆*Trdim5* strains as shown in (B). Significant differences (*t*‐test **p < 0.01) in conidia production between QM9414 and the ∆*Trdim5* strains were observed.

To investigate the *in vivo* function of *Tr*Dim5, the phenotypes of vegetative growth and conidiation were characterized in the ∆*Trdim5* strain. The ∆*Trdim5* and QM9414 strains were precultured on agar plates with minimal media until the plates were completely covered with diffused mycelia. An equal amount of mycelia was then removed from the plate and inoculated on agar plates with 1% (w/v) concentration of various tested carbon sources. Compared with the control strain QM9414, the hyphal growth of ∆*Trdim5* strain was significantly restricted after incubation at 30°C for 3 days, as shown in Figure 2A. Similarly, the sporulation of ∆*Trdim5* was severely reduced on the malt extract agar plate (Figure 2B), demonstrated by the dramatic decrease in conidia formation after incubation at 30°C for 5 days (Figure 2C).

### *Tr* *DIM5* knockout leads to enhanced sorbicillinoid secretion

**FIGURE 3:**
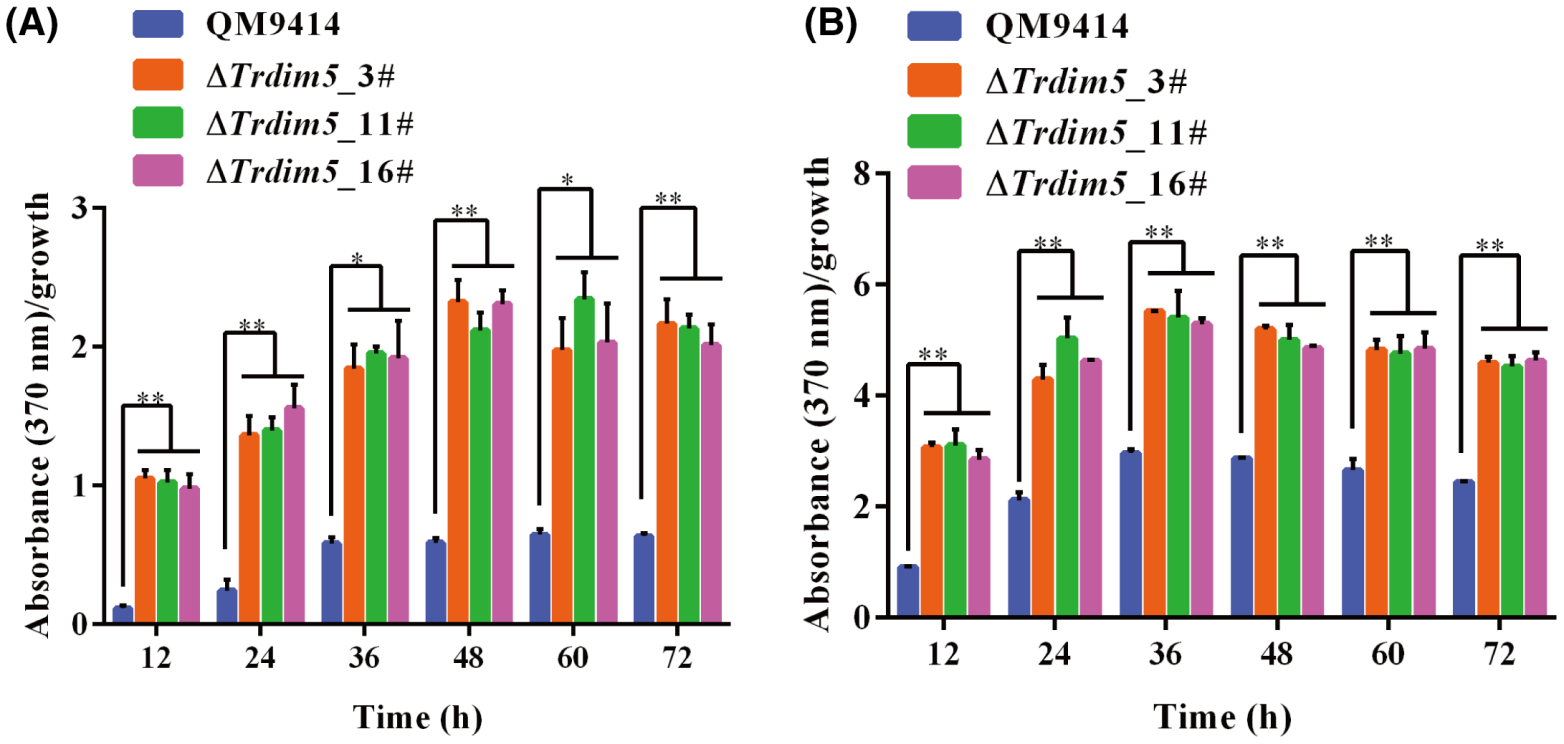
Deletion of *TrDIM5* leads to enhanced sorbicillinoid secretion. (A–B) Quantitative analysis of sorbicillinoid production by normalizing the absorbance at 370 nm of the supernatants from QM9414 and ∆*Trdim5* strains with growth on Avicel (A) or glucose (B). Significant differences (*t*‐test *p < 0.05 and **p < 0.01) in sorbicillinoid production between QM9414 and the ∆*Trdim5* strains were observed.

**FIGURE 4:**
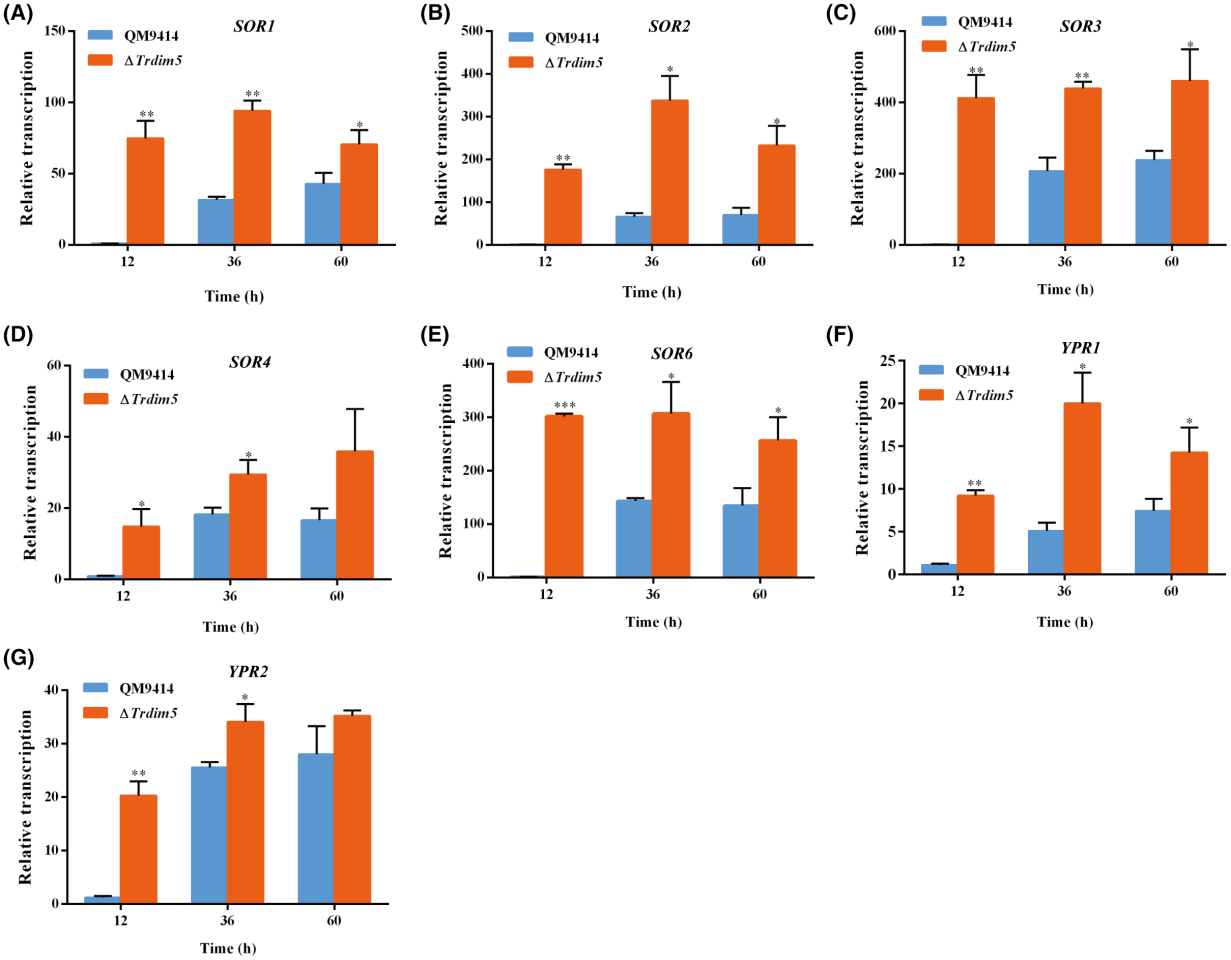
Knockout of *TrDIM5* resulted in a significant increase in the transcription of the *SOR* gene cluster. Transcription of *SOR1* (A), *SOR2* (B), *SOR3* (C), *SOR4* (D), *SOR6* (E), *YPR1* (F) and *YPR2* (G) was analysed by quantitative RT‐PCR after cultivation on 1% (w/v) Avicel for the indicated time periods. The *ACTIN* gene was used as a reference for normalization in all samples. Significant differences (*t*‐test *p < 0.05, **p < 0.01 and ***p < 0.001) were detected for the transcription of the above genes between QM9414 and ∆*Trdim5* strains.

To further evaluate the role of *Tr*Dim5 in sorbicillinoid synthesis, an equal amount of mycelia of the ∆*Trdim5* and control strains were inoculated into glucose‐ or Avicel‐containing medium. Consistently, dramatically enhanced production of the yellow pigment was observed in the ∆*Trdim5* strain regardless of whether Avicel or glucose was used as the sole carbon source (Figure 3A, B). Further examination of the mRNA abundance of the *SOR* genes by RT‐qPCR demonstrated a significant upregulation in the steady‐state *SOR* transcripts except *SOR5* in ∆*Trdim5* compared with QM9414 grown on Avicel‐containing medium (Figure 4A–G). The upregulation in *SOR* transcription without *Tr*Dim5 may well account for the drastically increased sorbicillinoid secretion in the ∆*Trdim5* strain. The silence of *SOR5* expression in either QM9414 or ∆*Trdim5* at all time points was consistent with earlier reports in other mutant strains that derived from *T. reesei* wild‐type QM6a (Derntl et al., 2016; Li et al., 2018). These results showed that *Tr*Dim5 has an essential role in the repression of sorbicillinoid biosynthesis at the transcriptional level.

### *Tr*Dim5 suppresses the expression of the SOR gene cluster rather than its adjacent genes

**FIGURE 5:**
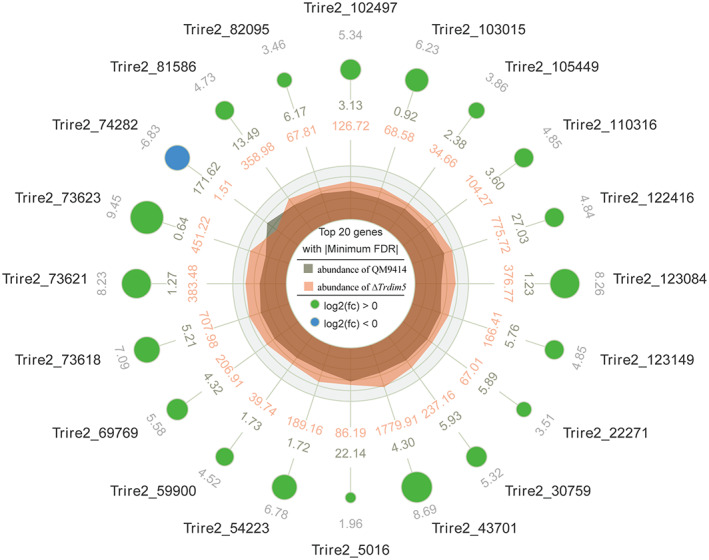
Analysis of the top 20 genes with the minimum FDR in DEGs using a radar map. Each dot represents a gene, and its expression level is indicated by different colours (green, upregulated; blue, downregulated). The dot size represents ‐1og_10_FDR. The numbers from outside to inside represent the gene ID, log_2_(FC), FPKM value in QM9414 and FPKM value in ∆*Trdim5*, respectively.

**FIGURE 6:**
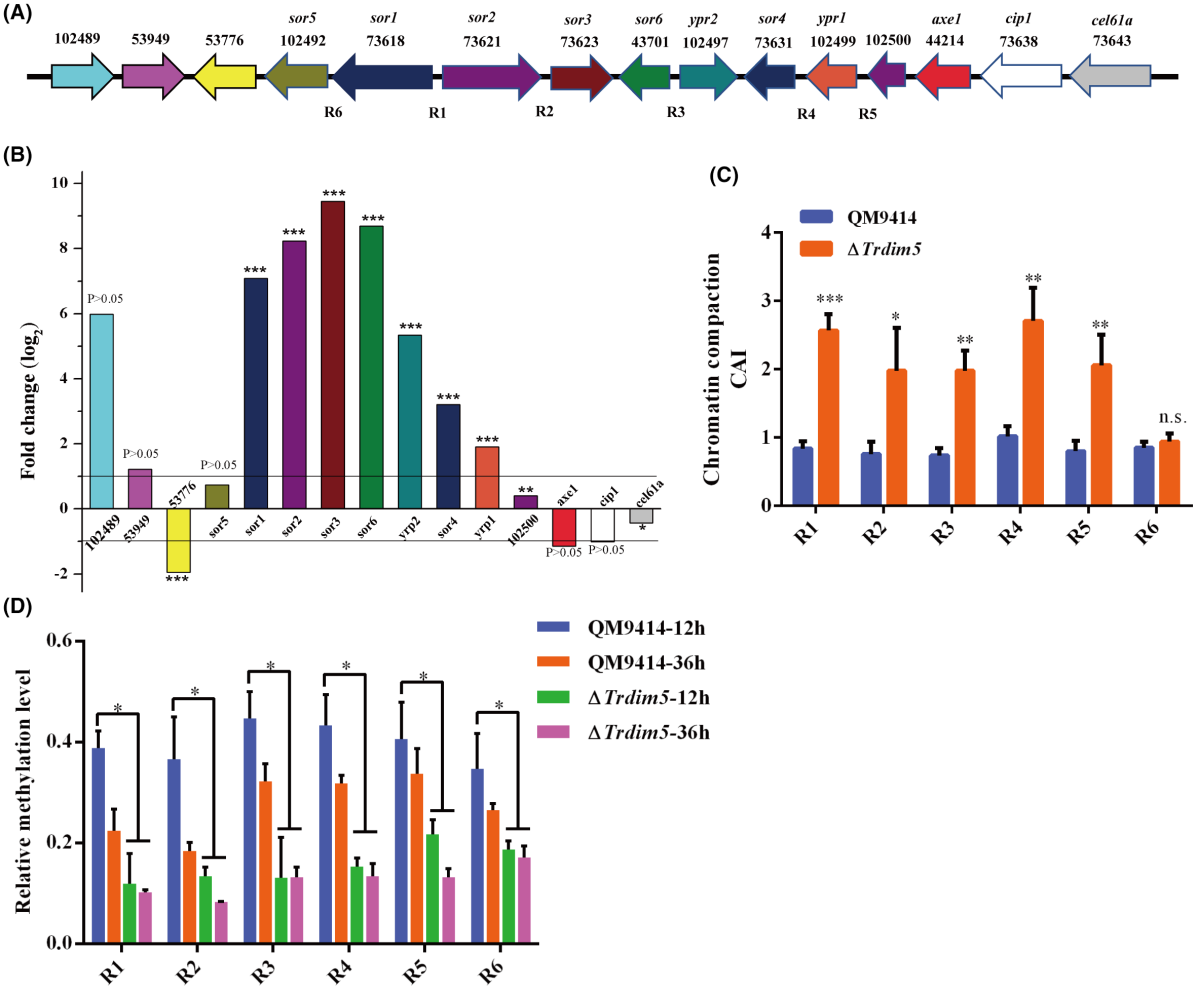
*Tr*Dim5‐mediated H3K9me3 was directly associated with *SOR* gene cluster repression. (A) Schematic representation of the *SOR* gene cluster and promoter region selected for CHART‐PCR and ChIP analyses. (B) The expression pattern of the *SOR* genes and their adjacent genes in the ∆*Trdim5* strain compared to QM9414 under Avicel‐inducing conditions. (C) Analysis of the chromatin accessibility at *SOR* gene promoters in the ∆*Trdim5* and QM9414 strains after 36 h of growth on Avicel. The chromatin accessibility index (CAI) was defined as CAI = 1/Ds/Dc, where Ds is the amount of intact DNA detected for each target region and Dc is the amount of intact DNA detected for the promoter region of the housekeeping gene *ACTIN*. CAI is depicted on the y‐axis, and the transcript level (FPKM) is depicted on the x‐axis. (D) Analysis of H3K9me3 enrichment using ChIP, followed by quantitative PCR at different chromatin regions and time points, as indicated.

To gain insight into how *Tr*Dim5 influences the transcription of the whole gene in *T. reesei*, RNA‐seq was performed to compare the transcript profiles of QM9414 and ∆*Trdim5* strains after incubation for 12 h with 1% Avicel as the sole carbon source. As a result, the entire read sequences were mapped with the reference genome of *T. reesei*, and 9758 unique transcripts were obtained (Data S1). Amongst these, 628 and 1879 genes were up‐ and downregulated, respectively (Data S2). GO enrichment analysis of the differentially expressed genes (DEGs) revealed that the most prevalently influenced genes (Top five GO terms) by *TrDIM5* knockout were mainly involved in helicase, ligase and transcription factor activities (Table S1, Data S3). Analysis of the top 20 genes with minimum false discovery rate (FDR) showed that five *SOR* genes (*YPR2*, *SOR1*, *SOR2*, *SOR3* and *SOR6*) were included as well as upregulated (Figure 5). Although other two *SOR* genes (*YPR1* and *SOR4*), were not assigned at the top 20 genes with the minimum FDR, they showed an increased expression in ∆*Trdim5*. Then, we retrieved an approximately 15 kb DNA region located on both sides of the *SOR* gene cluster. Three genes (*Trire2_102489*, *Trire2*_*53949* and *Trire2*_*53776*) were identified in the left chromatin arm and four genes (*Trire2_102500*, *AXE1*, *CIP1* and *CEL61a*) were identified in the right chromatin arm (Figure 6A). However, six of the above seven genes in ∆*Trdim5* exhibited either |log_2_FC| < 1 or FDR >0.05. Only the expression of *Trire2_53776* was significantly decreased in ∆*Trdim5* (Figure 6B). Collectively, these results showed that *Tr*Dim5 suppressed the expression of the *SOR* gene cluster but not its adjacent genes.

*Trichoderma reesei* genome contains 11 PKS‐encoding genes, 10 non‐ribosomal peptide synthetase (NRPS)‐encoding genes and two hybrid PKS‐NRPS (HPN)‐encoding genes. Based on RNA‐seq data, a detailed expression analysis of these secondary metabolic genes was performed. The results indicated that seven genes (*SOR1*, *SOR2*, *Trire2_106272*, *Trire2_65116*, *Trire2_81964*, *Trire2_58285 and Trire2_59315*) exhibited upregulation and two genes (*Trire2_82208* and *Trire2_59482*) exhibited downregulation (Table S2), suggesting that *Tr*Dim5 is an important regulator of secondary metabolism in *T. reesei*.

### *Tr*Dim5‐mediated epigenetic modification of H3K9me3 directly results in repression of the *SOR* gene cluster

As Dim5 orthologues play vital roles in transcriptional suppression of genes in eukaryotic cells, therefore, further insights were gained to know its regulatory mechanism on *SOR* gene cluster expression. Consequently, we investigated the chromatin packaging by applying chromatin accessibility real‐time PCR (CHART‐PCR) analysis at the promoter regions (R1–R6) of *SOR* genes (Figure 6A). Consistent with the higher expression, a more open chromatin status was found at the promoters of *SOR* genes in ∆*Trdim5* (Figure 6C).

To determine whether *Tr*Dim5‐mediated H3K9me3 directly or indirectly repressed the transcription of genes in the *SOR* gene cluster, chromatin immunoprecipitation (ChIP) assay was performed, followed by quantitative PCR (ChIP‐qPCR) using commercial H3K9me3 antibody to determine the correlation between *Tr*Dim5‐mediated H3K9me3 modification and transcription of *SOR* genes. In the control strain, the H3K9me3 signal was enriched at the R1–R6 regions at 12 h which was apparently decreased at 36 h along with *SOR* gene expression (Figures 4, 6). However, compared with the control strain, the R1–R6 regions of ∆*Trdim5* showed low‐level enrichment regardless of the incubation duration (Figure 6D). These data showed that *Tr*Dim5‐mediated H3K9me3 directly repressed sorbicillinoid biosynthesis and thus suggested its considerable potential to improve sorbicillinoid production in *T. reesei*.

### Upregulation of the *SOR* gene cluster in ∆*Trdim5* relies on the transcriptional activator Ypr1

**FIGURE 7:**
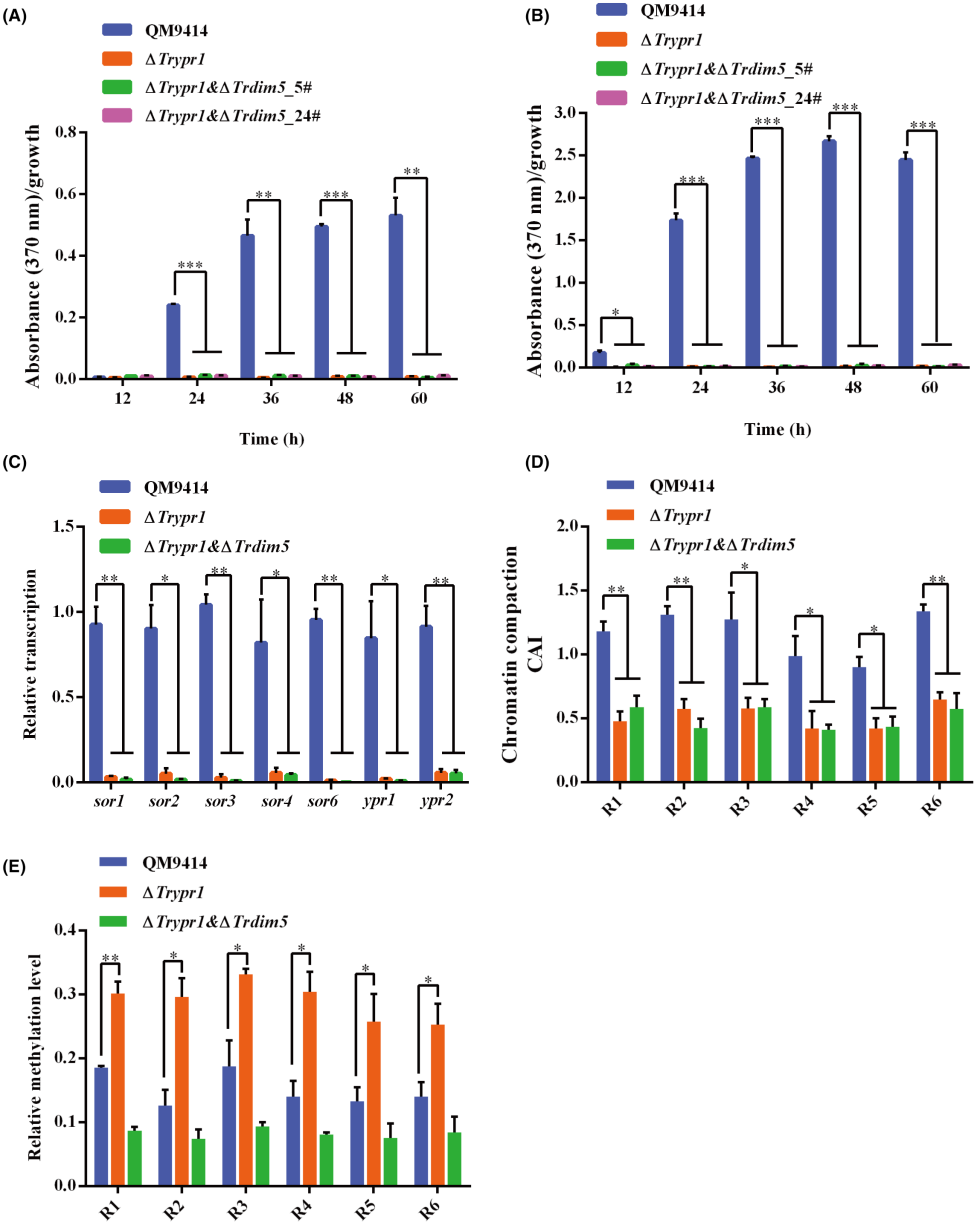
Upregulated expression of the *SOR* gene cluster in ∆*Trdim5* depends on the activator Ypr1. (A–B) Quantitative analysis of sorbicillinoid production by normalizing the absorbance at 370 nm of the supernatants from QM9414, ∆*Trypr1* and the two independent ∆*Trypr1* & ∆*Trdim5* strains using growth on Avicel (A) or glucose (B). (C) Transcription of *SOR* genes was analysed by quantitative RT‐PCR after cultivation on 1% (w/v) Avicel for 36 h. (D–E) Analysis of the chromatin accessibility (D) and H3K9me3 enrichment (E) at *SOR* gene promoters in the ∆*Trypr1*, ∆*Trypr1* & ∆*Trdim5* and QM9414 strains after 36 h of growth on Avicel.

Two transcription factors (Ypr1 and Ypr2) that are encoded in the *SOR* cluster have been shown to regulate the expression of the *SOR* genes (Derntl et al., 2016). Ypr1 is considered as a transcriptional activator for the expression of most genes within the *SOR* cluster, but the underlying mechanism through which Ypr1 regulates the *SOR* gene cluster is still unclear. To explore the possible link between *Tr*Dim5 and Ypr1 in the coordination of *SOR* gene cluster expression, a double knockout of *Trdim5* and *Trypr1* was developed in *T. reesei*. Subsequently, equal amounts of mycelia of the ∆*Trypr1*, ∆*Trypr1* & ∆*Trdim5*, and QM9414 were inoculated into 1% glucose‐ or Avicel‐containing medium to assess their capacity of sorbicillinoid synthesis. In contrast to ∆*Trdim5*, both the ∆*Trypr1* and ∆*Trypr1* & ∆*Trdim5* strains showed the abolition of sorbicillinoid synthesis regardless of whether glucose or Avicel was used as the sole carbon source (Figure 7A,B). Consistent with the absence of sorbicillinoid secretion in ∆*Trypr1* & ∆*Trdim5* mutant strains, the transcription of *SOR* genes was hardly detected (Figure 7C), indicating that Ypr1 is indispensable for the expression of the *SOR* gene cluster.

Compared with the control strain QM9414, CHART‐PCR analysis showed a more compact chromatin structure at the promoter regions of *SOR* genes in the ∆*Trypr1* and ∆*Trypr1* & ∆*Trdim5* strains (Figure 7D). Moreover, the promoter regions of *SOR* genes displayed higher enrichment of H3K9me3 modification in ∆*Trypr1* than QM9414 (Figure 7E), implying that Ypr1 was critical for relieving *Tr*Dim5‐mediated repression at *SOR* gene promoters. Conclusively, these results suggested that Ypr1 antagonizes *Tr*Dim5‐mediated H3K9me3 modification and thus contributes to forming a relatively more open local chromatin environment, which may further facilitate its binding and *SOR* gene expression.

## DISCUSSION

Fungi are a natural resource pool for bioactive compounds, which have benefitted the discovery of numerous secondary metabolic gene clusters in genome sequencing studies (Katz & Baltz, 2016). *T. reesei* is a well‐known industrial strain used for (hemi)cellulase and recombinant protein production. However, its secondary metabolism has received less attention. *T. reesei* can produce a large amount of yellow pigment which is identified to be a mixture of SMs known as sorbicillinoids with various bioactivities during cultivation (Salo et al., 2016; Seiboth et al., 2012). On the one hand, sorbicillinoids lead to complex downstream processes of protein production and thus increase the industrial costs. On the other hand, the potential bioactivities of sorbicillinoids have promoted investigations on their production and valuable applications (Meng et al., 2016). Recently, eight‐genes‐containing cluster which is responsible for sorbicillinoid biosynthesis is identified in *T. reesei* (Druzhinina et al., 2016) but this cluster is not found in its close relatives, *Trichoderma atroviride* and *Trichoderma virens* (Derntl et al., 2016). It has been clarified that the leading regulatory roles of two trans‐acting factors, Ypr1 and Ypr2, for the *SOR* gene cluster expression (Derntl et al., 2016; Hitzenhammer et al., 2019), thereby describing a strategy for controlling excess sorbicillinoid secretion during the production of enzymes or other proteins and for developing high‐yield strains of sorbicillinoid biosynthesis. However, the environmental cues and regulatory mechanisms for sorbicillinoid biosynthesis remain largely unknown.

Genomic sequence analyses revealed that secondary metabolic gene clusters in several fungi are generally located in the telomere or heterochromatin region, implying that these clusters' regulation is closely related to the chromatin status (Strauss & Reyes‐Dominguez, 2011). In eukaryotic cells, repressive histone modification H3K9me3 frequently coexists with DNA methylation and is involved in heterochromatin establishment and maintenance (Jackson et al., 2002; Rountree & Selker, 2010). Such a cross‐talk between H3K9me3 and DNA methylation has been elucidated in the heterochromatin establishment of *Neurospora crassa* (Tamaru et al., 2003). In this respect, Dim5 mediates H3K9me3 modification which is further recognized by the chromodomain of heterochromatin protein 1 (Hp1). The recruited Hp1 brings in DNA methyltransferase Dim2 which forms a stable complex with Hp1 to induce DNA methylation (Honda & Selker, 2008; Jacobs & Khorasanizadeh, 2002). In addition to Swi6 (the Hp1 orthologue in *S*. *pombe*), *Sp*Clr4 also has a chromodomain at its N‐terminus which is critical for the spread of H3K9me3 modification in establishing a repressive chromatin (Zhang et al., 2008). Unlike *Sp*Clr4, none of the known Clr4 orthologues from filamentous fungi including *T. reesei* have been found to include this chromodomain, implying that a different mode of H3K9me3 spread in chromatin exists in filamentous fungi.

In *S*. *pombe*, only one histone methyltransferase is responsible for the mono‐, di‐ and tri‐methylation of H3K9 (Kusevic et al., 2017). This study also verified that *TrDIM5* knockout nearly abolished the H3K9me3 modification, implying that *Tr*Dim5 represents a major methyltransferase of H3K9 in *T. reesei*. However, there is a possibility that other proteins with weaker H3K9 methyltransferase activity exist in *T. reesei*. Of note, deletion of *TrDIM5* impaired vegetative growth and conidiation, whereas the ∆*Trdim5* strain displayed enhanced sorbicillinoid production. It has been corroborated that *T. reesei SOR* genes are expressed during fast vegetative growth and antagonism against other fungi (Druzhinina et al., 2016). It is thus highly unlikely that the enhanced sorbicillinoid biosynthesis results from the impaired vegetative growth. Similarly, increased *SOR* gene expression has been also found in our recent studies with a chromatin remodeller whose absence compromised growth (Cao et al., 2022). Nevertheless, the impaired vegetative growth could be a mimicry of antagonism when confronted with other fungi that may evoke a self‐protection mechanism to promote sorbicillinoid biosynthesis in the absence of *Tr*Dim5.

In filamentous fungi, heterochromatin is frequently associated with secondary metabolic gene silencing. Interfering with heterochromatin establishment or maintenance has been proven to be effective in relieving the repression of SMs production (Reyes‐Dominguez et al., 2010; 2012). The results of this study showed that with *TrDIM5* knockout, the expression of *SOR* genes except *SOR5* was elevated along with a more open chromatin at their promoters. *SOR5* encodes a short‐chain dehydrogenase/reductase, which is only found in some *Trichoderma* species. In *Penicillium rubens*, an orthologue of *SOR5* is present in the genome but not located in the vicinity of the *SOR* gene cluster (Druzhinina et al., 2016). In *Acremonium chrysogenum*, a gene of unknown function is found at the position of *SOR5* (Druzhinina et al., 2016). Keeping this in view, there is a possibility that *SOR5* does not belong to the *SOR* gene cluster. Nevertheless, an obvious enrichment of H3K9me3 modification was detected at the *SOR5* promoter in the control strain. We speculated that the *Tr*Dim5‐mediated epigenetic modification extends to the adjacent nucleosomes of the *SOR* gene cluster by a yet unknown mechanism.

In most filamentous fungi, the putative methyltransferase LaeA is a vital secondary metabolic regulator that responds to environmental cues, such as light and temperature (Bayram et al., 2008; Hitzenhammer et al., 2019). For example, the deletion of *laeA* causes a loss of several SMs (sterigmatocystin, penicillin and terrequinone A) in *A. nidulans* (Bok et al., 2006). Similar results have been reported for the biosynthesis of lovastatin in *Aspergillus terreus*, aflatoxin in *Aspergillus flavus* and gliotoxin in *Aspergillus fumigatus* (Amare & Keller, 2014; Bok & Keller, 2004). In *T. reesei*, Lae1 (a LaeA orthologue) has already been identified as a crucial positive regulator of the cellulase and *SOR* gene expression (Druzhinina et al., 2016; Seiboth et al., 2012). However, our RNA‐seq results showed no significant difference in *LAE1* expression between ∆*Trdim5* and the control strain existed (Data S1). It seems that *LAE1* is not subjected to regulation of *Tr*Dim5 in *T. reesei*.

Further deletion of *TrDIM5* in ∆*Trypr1* strain resulted in an expression pattern similar to that of *SOR* genes in the ∆*Trypr1* strain. Compared with the control strain QM9414, the level of chromatin accessibility at *SOR* gene promoters in *∆Trypr1* and *∆Trypr1 & ∆Trdim5* strains was significantly reduced which well explained the compromised *SOR* transcription in these two strains. CHIP assays showed that H3K9me3 modification at *SOR* gene promoters in *∆Trypr1* was significantly higher than that of the control strain. Of note, although the H3K9me3 modification was even lower in the simultaneous absence of *Tr*Dim5 and *Tr*Ypr1, the observation that a lower chromatin accessibility existed rather indicates that the sole decrease in *Tr*Dim5‐mediated epigenetic modification at *SOR* gene promoters is not enough to elicit a readily open chromatin status without Ypr1 at *SOR* promoters to allow for the *SOR* gene transcription. The combined results, therefore, suggested that the initial Ypr1 binding somehow antagonizes *Tr*Dim5‐mediated H3K9me3 modification and in the meantime contributes to forming a relatively more open local chromatin environment, which may further facilitate its binding and *SOR* gene expression. These findings were helpful for clarifying the mechanism through which sorbicillinoid biosynthesis in *T. reesei* was regulated and provided a foundation to engineer a recombination strain with high‐yield sorbicillinoids based on complex regulatory networks.

## CONFLICT OF INTEREST

The authors declare that they have no competing interests.

## Supporting information


Data S1

Data S2

Data S3

Figure S1
Figure S2.
Figure S3.

Table S1
Table S2.
Table S3.

Table S2.

Table S3.

## Data Availability

The data that support the findings of this study are available within the article and its supporting information. Requests for raw data should be addressed to the corresponding author.
