# Supplementary material for: A histone H3K9 methyltransferase Dim5 mediates repression of sorbicillinoid biosynthesis in Trichoderma reesei
Source: Microb Biotechnol. 2022 Aug 3;15(10):2533–46. doi: 10.1111/1751-7915.14103 (PMC9518983; doi:10.1111/1751-7915.14103)
Supplement: Supplementary file 4 — Figure S1 Figure S2. Figure S3. [file MBT2-15-2533-s005.docx]

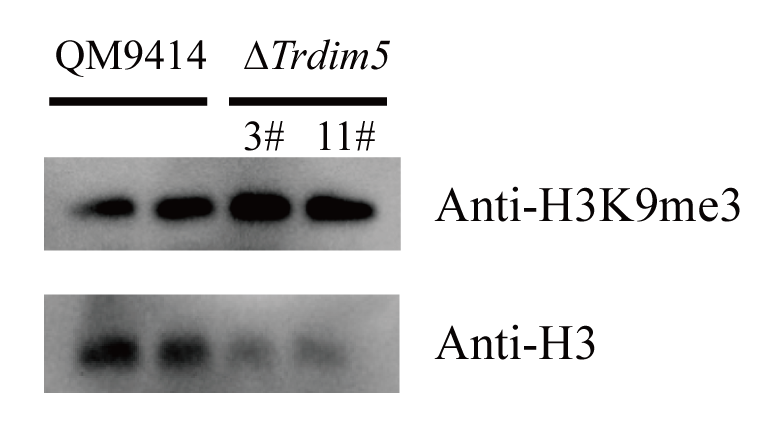


**Figure S1. Western blot analysis of H3K9me3 modification in QM9414 and ∆*Trdim5* strains, respectively.**


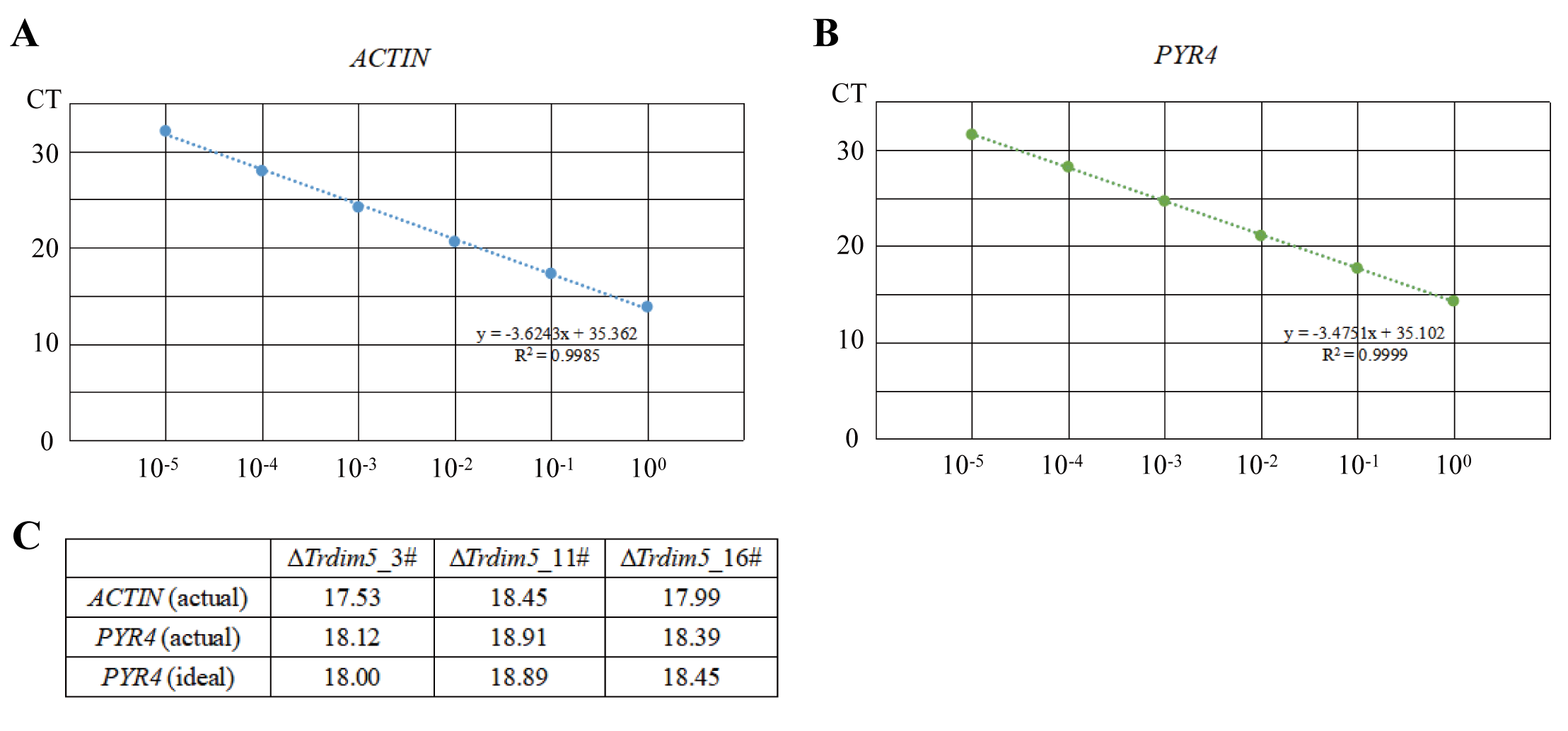


**Figure S2. Identification of the copy number of *Trdim5* knockout cassette by genomic qRCR in ∆*Trdim5* strain.** (A-B) *T. reesei* QM9414 genome was extracted and about 1 µg/µl genomic DNA was gradiently diluted to serve as a template for drawing of standard curves of *ACTIN* (A) and *PYR4* (B), respectively. (C) The actual CT values of *ACTIN* and *PYR4* were determinated by qPCR using genomic DNA of ∆*Trdim5* strain as a template. All the actual CT values in qPCR are derived from average value of three technical repetitions. Comparative analysis of ideal CT value calculated using standard curves and actual CT value measured by qPCR of *PYR4* was performed to confirm the copy number of *Trdim5* knockout cassette in ∆*Trdim5* strain. One copy of *Trdim5* knockout cassette in ∆*Trdim5* strain mean the ratio of actual CT value to ideal CT value of *PYR4* is close to 100%.


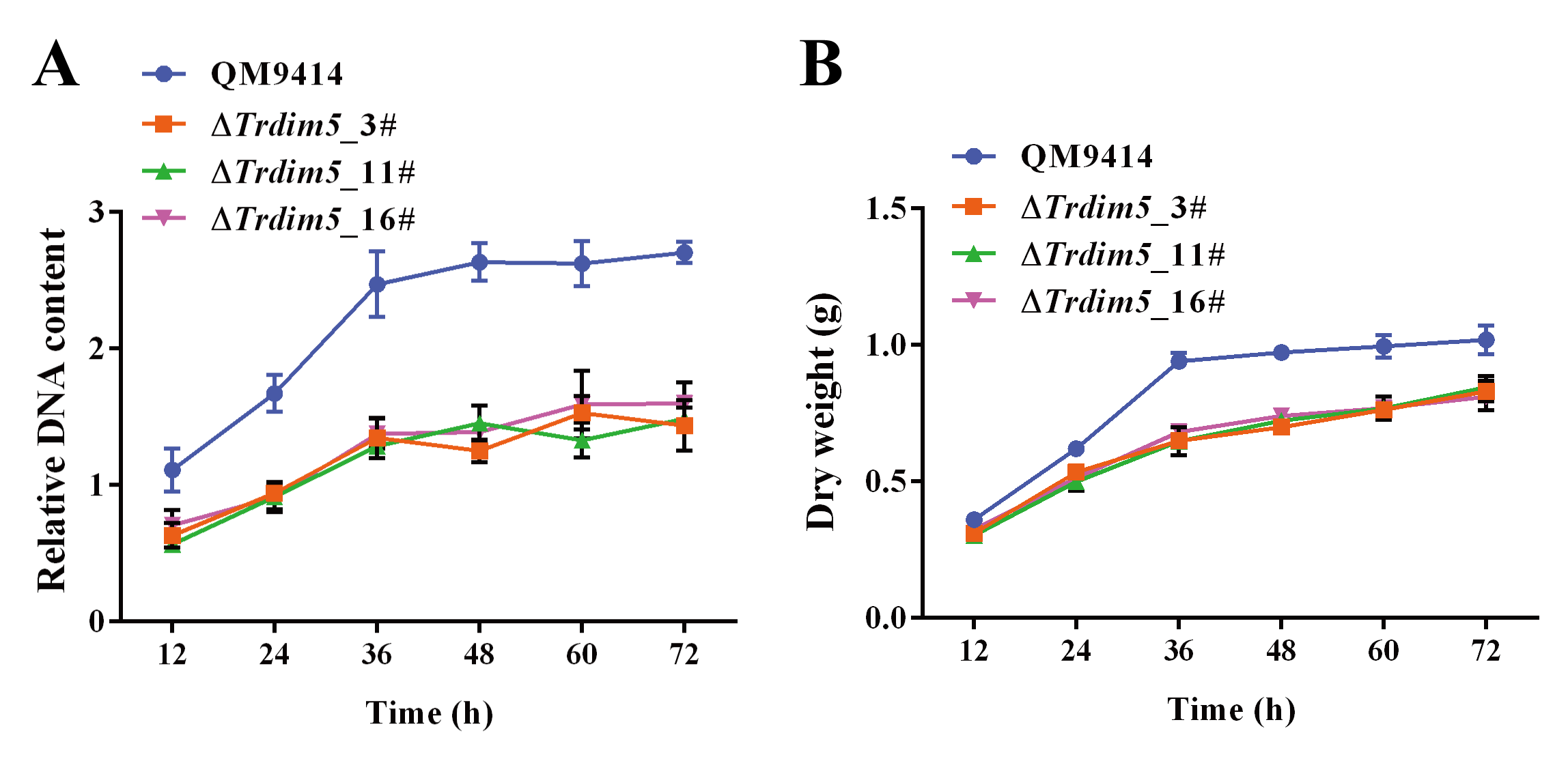


**Figure S3.** **Growth measurement of QM9414 and ∆*Trcdim5* strains cultured in liquid culture with 1% (w/v) Avicel or glucose as the sole carbon source.** (A) The 2 mL fermentation broth was collected and centrifuged. Genomic DNA of the mycelia in the sediment was extracted and the content was measured to determine the growth, with the sample of QM9414 at 12 h as the reference. (B) Mycelia cultured on glucose were filtrated on filter paper, dried at 70 °C for 48 h, and then weighed.
