# Supplementary material for: A histone H3K9 methyltransferase Dim5 mediates repression of sorbicillinoid biosynthesis in Trichoderma reesei
Source: Microb Biotechnol. 2022 Aug 3;15(10):2533–46. doi: 10.1111/1751-7915.14103 (PMC9518983; doi:10.1111/1751-7915.14103)
Supplement: Supplementary file 5 — Table S1 Table S2. Table S3. [file MBT2-15-2533-s002.docx]

| **Table S1 GO enrichment analysis of DEGs with the threshold of FDR<0.05** | | | | | | |
| --- | --- | --- | --- | --- | --- | --- |
| # | GO ID | Description | DEGs | All | proportion | FDR |
| **Molecular Function** | | | | | | |
| 1 | ^#^[GO:1901363](file:///C:\\Users\\zhangfeng\\Desktop\\clr4%E6%96%87%E7%AB%A0\\GDER20060192-sup_2%E8%BD%AC%E5%BD%95%E7%BB%84%E5%8A%A0ID\\GDER20060192-Trichoderma_reesei-9_RNAseq-result\\7.GroupDiffExpression\\enrich\\GO\\QM-vs-clr4.F.html#gene1) | heterocyclic compound binding | 493 | 1417 | 0.35 | 0.000000 |
| 2 | ^#^[GO:0097159](file:///C:\\Users\\zhangfeng\\Desktop\\clr4%E6%96%87%E7%AB%A0\\GDER20060192-sup_2%E8%BD%AC%E5%BD%95%E7%BB%84%E5%8A%A0ID\\GDER20060192-Trichoderma_reesei-9_RNAseq-result\\7.GroupDiffExpression\\enrich\\GO\\QM-vs-clr4.F.html#gene2) | organic cyclic compound binding | 506 | 1465 | 0.35 | 0.000000 |
| 3 | ^#^[GO:0001882](file:///C:\\Users\\zhangfeng\\Desktop\\clr4%E6%96%87%E7%AB%A0\\GDER20060192-sup_2%E8%BD%AC%E5%BD%95%E7%BB%84%E5%8A%A0ID\\GDER20060192-Trichoderma_reesei-9_RNAseq-result\\7.GroupDiffExpression\\enrich\\GO\\QM-vs-clr4.F.html#gene3) | nucleoside binding | 242 | 616 | 0.39 | 0.000000 |
| 4 | ^#^[GO:0001883](file:///C:\\Users\\zhangfeng\\Desktop\\clr4%E6%96%87%E7%AB%A0\\GDER20060192-sup_2%E8%BD%AC%E5%BD%95%E7%BB%84%E5%8A%A0ID\\GDER20060192-Trichoderma_reesei-9_RNAseq-result\\7.GroupDiffExpression\\enrich\\GO\\QM-vs-clr4.F.html#gene4) | purine nucleoside binding | 239 | 610 | 0.39 | 0.000000 |
| 5 | ^#^[GO:0032550](file:///C:\\Users\\zhangfeng\\Desktop\\clr4%E6%96%87%E7%AB%A0\\GDER20060192-sup_2%E8%BD%AC%E5%BD%95%E7%BB%84%E5%8A%A0ID\\GDER20060192-Trichoderma_reesei-9_RNAseq-result\\7.GroupDiffExpression\\enrich\\GO\\QM-vs-clr4.F.html#gene5) | purine ribonucleoside binding | 239 | 610 | 0.39 | 0.000000 |
| 6 | ^#^[GO:0097367](file:///C:\\Users\\zhangfeng\\Desktop\\clr4%E6%96%87%E7%AB%A0\\GDER20060192-sup_2%E8%BD%AC%E5%BD%95%E7%BB%84%E5%8A%A0ID\\GDER20060192-Trichoderma_reesei-9_RNAseq-result\\7.GroupDiffExpression\\enrich\\GO\\QM-vs-clr4.F.html#gene6) | carbohydrate derivative binding | 252 | 650 | 0.39 | 0.000000 |
| 7 | ^#^[GO:0032549](file:///C:\\Users\\zhangfeng\\Desktop\\clr4%E6%96%87%E7%AB%A0\\GDER20060192-sup_2%E8%BD%AC%E5%BD%95%E7%BB%84%E5%8A%A0ID\\GDER20060192-Trichoderma_reesei-9_RNAseq-result\\7.GroupDiffExpression\\enrich\\GO\\QM-vs-clr4.F.html#gene7) | ribonucleoside binding | 239 | 611 | 0.39 | 0.000000 |
| 8 | ^#^[GO:0036094](file:///C:\\Users\\zhangfeng\\Desktop\\clr4%E6%96%87%E7%AB%A0\\GDER20060192-sup_2%E8%BD%AC%E5%BD%95%E7%BB%84%E5%8A%A0ID\\GDER20060192-Trichoderma_reesei-9_RNAseq-result\\7.GroupDiffExpression\\enrich\\GO\\QM-vs-clr4.F.html#gene8) | small molecule binding | 303 | 830 | 0.37 | 0.000001 |
| 9 | ^#^[GO:0005488](file:///C:\\Users\\zhangfeng\\Desktop\\clr4%E6%96%87%E7%AB%A0\\GDER20060192-sup_2%E8%BD%AC%E5%BD%95%E7%BB%84%E5%8A%A0ID\\GDER20060192-Trichoderma_reesei-9_RNAseq-result\\7.GroupDiffExpression\\enrich\\GO\\QM-vs-clr4.F.html#gene9) | binding | 802 | 2531 | 0.32 | 0.000001 |
| 10 | ^#^[GO:0003676](file:///C:\\Users\\zhangfeng\\Desktop\\clr4%E6%96%87%E7%AB%A0\\GDER20060192-sup_2%E8%BD%AC%E5%BD%95%E7%BB%84%E5%8A%A0ID\\GDER20060192-Trichoderma_reesei-9_RNAseq-result\\7.GroupDiffExpression\\enrich\\GO\\QM-vs-clr4.F.html#gene10) | nucleic acid binding | 226 | 633 | 0.36 | 0.000479 |
| 11 | ^#^[GO:0004386](file:///C:\\Users\\zhangfeng\\Desktop\\clr4%E6%96%87%E7%AB%A0\\GDER20060192-sup_2%E8%BD%AC%E5%BD%95%E7%BB%84%E5%8A%A0ID\\GDER20060192-Trichoderma_reesei-9_RNAseq-result\\7.GroupDiffExpression\\enrich\\GO\\QM-vs-clr4.F.html#gene11)* | helicase activity | 26 | 47 | 0.55 | 0.004398 |
| 12 | ^#^[GO:0017111](file:///C:\\Users\\zhangfeng\\Desktop\\clr4%E6%96%87%E7%AB%A0\\GDER20060192-sup_2%E8%BD%AC%E5%BD%95%E7%BB%84%E5%8A%A0ID\\GDER20060192-Trichoderma_reesei-9_RNAseq-result\\7.GroupDiffExpression\\enrich\\GO\\QM-vs-clr4.F.html#gene12) | nucleoside-triphosphatase activity | 114 | 302 | 0.38 | 0.006912 |
| 13 | ^#^[GO:0016875](file:///C:\\Users\\zhangfeng\\Desktop\\clr4%E6%96%87%E7%AB%A0\\GDER20060192-sup_2%E8%BD%AC%E5%BD%95%E7%BB%84%E5%8A%A0ID\\GDER20060192-Trichoderma_reesei-9_RNAseq-result\\7.GroupDiffExpression\\enrich\\GO\\QM-vs-clr4.F.html#gene13)* | ligase activity, forming carbon-oxygen bonds | 25 | 46 | 0.54 | 0.006912 |
| 14 | ^#^[GO:0016876](file:///C:\\Users\\zhangfeng\\Desktop\\clr4%E6%96%87%E7%AB%A0\\GDER20060192-sup_2%E8%BD%AC%E5%BD%95%E7%BB%84%E5%8A%A0ID\\GDER20060192-Trichoderma_reesei-9_RNAseq-result\\7.GroupDiffExpression\\enrich\\GO\\QM-vs-clr4.F.html#gene14)* | ligase activity, forming aminoacyl-tRNA and related compounds | 25 | 46 | 0.54 | 0.006912 |
| 15 | ^#^[GO:0004812](file:///C:\\Users\\zhangfeng\\Desktop\\clr4%E6%96%87%E7%AB%A0\\GDER20060192-sup_2%E8%BD%AC%E5%BD%95%E7%BB%84%E5%8A%A0ID\\GDER20060192-Trichoderma_reesei-9_RNAseq-result\\7.GroupDiffExpression\\enrich\\GO\\QM-vs-clr4.F.html#gene15)* | aminoacyl-tRNA ligase activity | 24 | 44 | 0.5 | 0.008061 |
| 16 | ^#^[GO:0016818](file:///C:\\Users\\zhangfeng\\Desktop\\clr4%E6%96%87%E7%AB%A0\\GDER20060192-sup_2%E8%BD%AC%E5%BD%95%E7%BB%84%E5%8A%A0ID\\GDER20060192-Trichoderma_reesei-9_RNAseq-result\\7.GroupDiffExpression\\enrich\\GO\\QM-vs-clr4.F.html#gene16) | hydrolase activity, acting on acid anhydrides, in phosphorus-containing anhydrides | 119 | 324 | 0.37 | 0.014734 |
| 17 | ^#^[GO:0016817](file:///C:\\Users\\zhangfeng\\Desktop\\clr4%E6%96%87%E7%AB%A0\\GDER20060192-sup_2%E8%BD%AC%E5%BD%95%E7%BB%84%E5%8A%A0ID\\GDER20060192-Trichoderma_reesei-9_RNAseq-result\\7.GroupDiffExpression\\enrich\\GO\\QM-vs-clr4.F.html#gene17) | hydrolase activity, acting on acid anhydrides | 120 | 328 | 0.37 | 0.015679 |
| 18 | ^#^[GO:0001071](file:///C:\\Users\\zhangfeng\\Desktop\\clr4%E6%96%87%E7%AB%A0\\GDER20060192-sup_2%E8%BD%AC%E5%BD%95%E7%BB%84%E5%8A%A0ID\\GDER20060192-Trichoderma_reesei-9_RNAseq-result\\7.GroupDiffExpression\\enrich\\GO\\QM-vs-clr4.F.html#gene18)* | nucleic acid binding transcription factor activity | 31 | 65 | 0.48 | 0.020615 |
| 19 | ^#^[GO:0016874](file:///C:\\Users\\zhangfeng\\Desktop\\clr4%E6%96%87%E7%AB%A0\\GDER20060192-sup_2%E8%BD%AC%E5%BD%95%E7%BB%84%E5%8A%A0ID\\GDER20060192-Trichoderma_reesei-9_RNAseq-result\\7.GroupDiffExpression\\enrich\\GO\\QM-vs-clr4.F.html#gene19) | ligase activity | 49 | 116 | 0.42 | 0.023620 |
| 20 | [GO:0016462](file:///C:\\Users\\zhangfeng\\Desktop\\clr4%E6%96%87%E7%AB%A0\\GDER20060192-sup_2%E8%BD%AC%E5%BD%95%E7%BB%84%E5%8A%A0ID\\GDER20060192-Trichoderma_reesei-9_RNAseq-result\\7.GroupDiffExpression\\enrich\\GO\\QM-vs-clr4.F.html#gene20) | pyrophosphatase activity | 116 | 321 | 0.36 | 0.028219 |
| **Biological Process** | | | | | | |
| 1 | ^#^[GO:0050789](file:///C:\\Users\\zhangfeng\\Desktop\\clr4%E6%96%87%E7%AB%A0\\GDER20060192-sup_2%E8%BD%AC%E5%BD%95%E7%BB%84%E5%8A%A0ID\\GDER20060192-Trichoderma_reesei-9_RNAseq-result\\7.GroupDiffExpression\\enrich\\GO\\QM-vs-clr4.P.html#gene1) | regulation of biological process | 393 | 1159 | 0.34 | 0.008999 |
| 2 | [GO:0065007](file:///C:\\Users\\zhangfeng\\Desktop\\clr4%E6%96%87%E7%AB%A0\\GDER20060192-sup_2%E8%BD%AC%E5%BD%95%E7%BB%84%E5%8A%A0ID\\GDER20060192-Trichoderma_reesei-9_RNAseq-result\\7.GroupDiffExpression\\enrich\\GO\\QM-vs-clr4.P.html#gene2) | biological regulation | 424 | 1283 | 0.33 | 0.032895 |
| 3 | [GO:0019222](file:///C:\\Users\\zhangfeng\\Desktop\\clr4%E6%96%87%E7%AB%A0\\GDER20060192-sup_2%E8%BD%AC%E5%BD%95%E7%BB%84%E5%8A%A0ID\\GDER20060192-Trichoderma_reesei-9_RNAseq-result\\7.GroupDiffExpression\\enrich\\GO\\QM-vs-clr4.P.html#gene3) | regulation of metabolic process | 295 | 867 | 0.34 | 0.032895 |
| 4 | [GO:0022402](file:///C:\\Users\\zhangfeng\\Desktop\\clr4%E6%96%87%E7%AB%A0\\GDER20060192-sup_2%E8%BD%AC%E5%BD%95%E7%BB%84%E5%8A%A0ID\\GDER20060192-Trichoderma_reesei-9_RNAseq-result\\7.GroupDiffExpression\\enrich\\GO\\QM-vs-clr4.P.html#gene4) | cell cycle process | 132 | 352 | 0.38 | 0.032895 |
| 5 | [GO:0051276](file:///C:\\Users\\zhangfeng\\Desktop\\clr4%E6%96%87%E7%AB%A0\\GDER20060192-sup_2%E8%BD%AC%E5%BD%95%E7%BB%84%E5%8A%A0ID\\GDER20060192-Trichoderma_reesei-9_RNAseq-result\\7.GroupDiffExpression\\enrich\\GO\\QM-vs-clr4.P.html#gene5) | chromosome organization | 110 | 285 | 0.39 | 0.032895 |
| 6 | [GO:0050794](file:///C:\\Users\\zhangfeng\\Desktop\\clr4%E6%96%87%E7%AB%A0\\GDER20060192-sup_2%E8%BD%AC%E5%BD%95%E7%BB%84%E5%8A%A0ID\\GDER20060192-Trichoderma_reesei-9_RNAseq-result\\7.GroupDiffExpression\\enrich\\GO\\QM-vs-clr4.P.html#gene6) | regulation of cellular process | 337 | 1009 | 0.33 | 0.032895 |
| 7 | [GO:0071840](file:///C:\\Users\\zhangfeng\\Desktop\\clr4%E6%96%87%E7%AB%A0\\GDER20060192-sup_2%E8%BD%AC%E5%BD%95%E7%BB%84%E5%8A%A0ID\\GDER20060192-Trichoderma_reesei-9_RNAseq-result\\7.GroupDiffExpression\\enrich\\GO\\QM-vs-clr4.P.html#gene7) | cellular component organization or biogenesis | 359 | 1083 | 0.33 | 0.032895 |
| 8 | [GO:0006418](file:///C:\\Users\\zhangfeng\\Desktop\\clr4%E6%96%87%E7%AB%A0\\GDER20060192-sup_2%E8%BD%AC%E5%BD%95%E7%BB%84%E5%8A%A0ID\\GDER20060192-Trichoderma_reesei-9_RNAseq-result\\7.GroupDiffExpression\\enrich\\GO\\QM-vs-clr4.P.html#gene8) | tRNA aminoacylation for protein translation | 24 | 43 | 0.56 | 0.032895 |
| 9 | [GO:0043038](file:///C:\\Users\\zhangfeng\\Desktop\\clr4%E6%96%87%E7%AB%A0\\GDER20060192-sup_2%E8%BD%AC%E5%BD%95%E7%BB%84%E5%8A%A0ID\\GDER20060192-Trichoderma_reesei-9_RNAseq-result\\7.GroupDiffExpression\\enrich\\GO\\QM-vs-clr4.P.html#gene9) | amino acid activation | 26 | 48 | 0.54 | 0.032895 |
| 10 | [GO:0043039](file:///C:\\Users\\zhangfeng\\Desktop\\clr4%E6%96%87%E7%AB%A0\\GDER20060192-sup_2%E8%BD%AC%E5%BD%95%E7%BB%84%E5%8A%A0ID\\GDER20060192-Trichoderma_reesei-9_RNAseq-result\\7.GroupDiffExpression\\enrich\\GO\\QM-vs-clr4.P.html#gene10) | tRNA aminoacylation | 26 | 48 | 0.54 | 0.032895 |
| 11 | [GO:0006996](file:///C:\\Users\\zhangfeng\\Desktop\\clr4%E6%96%87%E7%AB%A0\\GDER20060192-sup_2%E8%BD%AC%E5%BD%95%E7%BB%84%E5%8A%A0ID\\GDER20060192-Trichoderma_reesei-9_RNAseq-result\\7.GroupDiffExpression\\enrich\\GO\\QM-vs-clr4.P.html#gene11) | organelle organization | 218 | 627 | 0.35 | 0.037176 |

^#^Top 20 GO terms with minimum FDR

^*^Top five GO terms are most prevalently influenced by *TrDIM5* knockout
