# Supplementary material for: A histone H3K9 methyltransferase Dim5 mediates repression of sorbicillinoid biosynthesis in Trichoderma reesei
Source: Microb Biotechnol. 2022 Aug 3;15(10):2533–46. doi: 10.1111/1751-7915.14103 (PMC9518983; doi:10.1111/1751-7915.14103)
Supplement: Supplementary file 6 — Table S2. [file MBT2-15-2533-s004.docx]

| Gene ID | QM9414  FPKM | ∆*Trclr4*  FPKM | log2  (∆*Trclr4*/QM9414) | FDR | Category |
| --- | --- | --- | --- | --- | --- |
| 73618**^a^** | 5.21 | 707.98 | 7.09 | 0 | PKS |
| 65172 | 0.02 | 0.04 | 1 | 0.16 | PKS |
| 59482**^b^** | 20.67 | 2.16 | -3.26 | 1.86E-34 | PKS |
| 60118 | 5.16 | 7.10 | 0.46 | 2.62E-08 | PKS |
| 106272**^a^** | 7.17 | 31.02 | 2.11 | 2.26E-24 | PKS |
| 65891 | 0.76 | 0.75 | -0.019 | 0.008 | PKS |
| 65116**^a^** | 0.08 | 0.23 | 1.56 | 7.84E-05 | PKS |
| 81964**^a^** | 0.09 | 0.21 | 1.25 | 0.002 | PKS |
| 73621**^a^** | 1.27 | 383.48 | 8.23 | 1.24E-267 | PKS |
| 105804 | 0.0067 | 0.01 | 0.58 | 0.80 | PKS |
| 82208**^b^** | 9.68 | 1.94 | -2.32 | 8.59E-09 | PKS |
| 123786 | 19.46 | 13.35 | -0.54 | 0.51 | NRPS |
| 23171 | 9.91 | 6.56 | -0.60 | 0.67 | NRPS |
| 71005 | 0.17 | 0.057 | -1.58 | 0.29 | NRPS |
| 60458 | 0.47 | 0.14 | -1.76 | 0.07 | NRPS |
| 69946 | 39.89 | 67.97 | 0.77 | 3.61E-11 | NRPS |
| 68204 | 2.54 | 1.07 | -1.24 | 0.10 | NRPS |
| 81014 | 74.20 | 86.04 | 0.21 | 1.53E-12 | NRPS |
| 24586 | 0.001 | 0.0067 | 2.74 | 0.54 | NRPS |
| 67189 | 5.46 | 2.42 | -1.17 | 0.06 | NRPS |
| 60751 | 0.20 | 0.26 | 0.40 | 0.12 | NRPS |
| 58285**^a^** | 0.15 | 0.44 | 1.55 | 4.49E-12 | HPN |
| 59315**^a^** | 0.11 | 0.32 | 1.57 | 7.54E-09 | HPN |

**Table S2 Comparative analysis of the expression level of PKS-, NRPS-, and HPN-encoding genes in QM9414 and ∆*Trdim5* strains.**

**^a^**upregulated

**^b^**downregulated
