# Supplementary material for: A histone H3K9 methyltransferase Dim5 mediates repression of sorbicillinoid biosynthesis in Trichoderma reesei
Source: Microb Biotechnol. 2022 Aug 3;15(10):2533–46. doi: 10.1111/1751-7915.14103 (PMC9518983; doi:10.1111/1751-7915.14103)
Supplement: Supplementary file 7 — Table S3. [file MBT2-15-2533-s003.docx]

**Table S3 Primers used in this study**

| **Plasmid construction** | |
| --- | --- |
| *dim5*-up-F | 5′-TAGGGATAACAGGGTAATCGATGGCCCAGCGTAGAA-3′ |
| *dim5*-up-R | 5′-GGGGACAAGTTTGTACAAAAAAGCAGGCTAAATAACCTCCTGA  ATCAGTCGTA-3′ |
| *dim5*-down-F | 5′-GGGGACCACTTTGTACAAGAAAGCTGGGTACAAACCGCATCATCCAAC-3′ |
| *dim5*-down-R | 5′-ATTACCCTGTTATCCCTATCATGGCTGGCCTGATTT-3′ |
| **Genomic qPCR** |  |
| *actin*-F | 5′-CATCGTGGCAGCGGAGTTA-3′ |
| *actin*-R | 5′-TTGAAGAGGGCGAAGATAGACA-3′ |
| *pyr4*-F | 5′-GCCCGACGATGCCTTTAT-3′ |
| *pyr4*-R | 5′-GGCTTTCCACGCTGCTGA-3′ |
| **RT-qPCR** |  |
| *ypr1-*qF | 5′-GTTCTACACACGACTTCCCATG-3′ |
| *ypr1-*qR | 5′-CCAGCCACTGATGTCGTATCC-3′ |
| *ypr2-*qF | 5′-GCTGCTTGAACAAATGGGAG-3′ |
| *ypr2-*qR | 5′-GCACATTCTTGGAGGAGTCG-3′ |
| *sor1-*qF | 5′-GGCCTTTGTTCTTCATGACTCC-3′ |
| *sor1-*qR | 5′-GTGAGCCAAGGCATCTTCG-3′ |
| *sor2-*qF | 5′-AGCTACTCAACAACGTGACGC-3′ |
| *sor2-*qR | 5′-ATCCCACTGCTGCTCAGGTAC-3′ |
| *sor3-*qF | 5′-CTTCGTCTTGAGTGTTCCTCTG-3′ |
| *sor3-*qR | 5′-GGCAGCAACGATATAAGCGAG-3′ |
| *sor4-*qF | 5′-CCTGGTAGTGAGAAACACGG-3′ |
| *sor4-*qR | 5′-GGCCAACAGTCGGACATATC-3′ |
| *sor6-*qF | 5′-GTCTACATGTTCCTGGGAGCC-3′ |
| *sor6-*qR | 5′-CAATGCTAACAGCCCAGTGG-3′ |
| *actin*-qF | 5′-TGAGAGCGGTGGTATCCACG-3′ |
| *actin*-qR | 5′-GGTACCACCAGACATGACAATGTTG-3′ |
| **ChIP-qPCR**  **/CHART-PCR** |  |
| R1-F | 5′-CGCACTTGACGACTCCTT-3′ |
| R1-R | 5′-TCTTGCCATGACGAACCT-3′ |
| R2-F | 5′-GAGATTTCACAGTAAGGGTT-3′ |
| R2-R | 5′-GGGTAGTCATTTGCTGGT-3′ |
| R3-F | 5′-TGTCGTCGGCAAGTGATT-3′ |
| R3-R | 5′-CCTCGCCTCCCAGATAGA-3′ |
| R4-F | 5′-TTTTGGCTCCCTTAGTAGA-3′ |
| R4-R | 5′-CTGCGAGTCAAGAGTATCAC-3′ |
| R5-F | 5′-GCCTGGAAGTAGGATGAG-3′ |
| R5-R | 5′-TGAAACGGTAGGATAATGAG-3′ |
| R6_F | 5′-TTAGCCAGCCTGAGAAAG-3′ |
| R6_R | 5′-TACGGTGCGAACAGTGAT-3′ |
| P*actin*_F | 5′-CATCGTGGCAGCGGAGTTA-3′ |
| P*actin*_R | 5′-TTGAAGAGGGCGAAGATAGACA-3′ |
